# An atypical pneumonia

**DOI:** 10.1002/rcr2.407

**Published:** 2019-02-05

**Authors:** Benjamin Gerhardy

**Affiliations:** ^1^ Department of Thoracic Medicine The Prince Charles Hospital Brisbane Australia

**Keywords:** Glomerulonephritis, HIV, vasculitis

## Abstract

We describe a patient with underlying HIV presenting with progressive respiratory distress and acute renal failure. A unifying diagnosis of microscopic polyangiitis was made. Following immunosuppression induction with plasma exchange and intravenous corticosteroid and subsequent maintenance immunosuppression with intravenous cyclophosphamide in conjunction with renal replacement therapy he achieved remission. To our knowledge this is the first documented case of microscopic polyangiitis occurring in the context of underlying HIV, and raises interesting possibilities regarding the development of vasculitis in this patient.

## Introduction

Anti‐Neutrophil Cytoplasmic Antibody (ANCA)‐associated vasculitides are a rare condition with an unknown pathogenesis. The role of the ANCA‐antibody is considered pathologic, although whether they are a primary illness or epiphenomena is unclear. HIV, also an uncommon condition in the developed world, has been implicated in the development of vascular disease although such a link has not previously been described with microscopic polyangiitis (MPA). We describe a case of MPA occurring in the context of HIV and highlight the danger in anchoring bias with such an underlying diagnosis.

## Case Report

A 70‐year‐old Caucasian male presented to emergency with 10 days of dry cough, dyspnoea, and fatigue. His respiratory rate was 18 per minute with saturations of 93% on 2 L of nasal prong oxygen, heart rate 75 beats per minute and in sinus rhythm, blood pressure 104/60 and was afebrile. Examination demonstrated globally reduced air entry with bibasal crackles with no other pertinent findings.

His medical history included HIV diagnosed in 1997 and well controlled with combination anti‐retroviral therapy (sequential viral loads undetectable and CD4 >500/mm^3^) and Barrett's oesophagus. His surgical history included a trans‐urethral prostate resection for hypertrophy as well as a left total knee replacement and a decompression for spinal stenosis. Medications were allopurinol, rosuvastatin, atazanavir, ritonavir, lamivudine, zidovudine, and pantoprazole. He had a 40 pack‐year smoking history (quit 15 years ago), minimal alcohol, and no illicit substance history. He worked in an office, was an active swimmer, and not in a relationship. Vaccines were up to date.

Initial investigations revealed a haemoglobin of 115 g/L, neutrophils 8.19 × 10^9^/L, lymphocyte 0.49 × 10^9^/L, eosinophils 0.17 × 10^9^/L, and platelets 390 × 10^9^/L. Creatinine was 441 micromol/L (86 six months prior), urea 25.4 mg/dL, and C‐reactive protein 148 mg/L. Chest film demonstrated diffuse bilateral infiltrates predominantly in the lower zones. Urinalysis demonstrated >500 erythrocytes, 10 leucocytes, and no bacteria.

With a provisional diagnosis of pneumonia he was commenced on ceftriaxone, azithromycin, and oxygen. Intravenous fluids continued.

The following day his creatinine increased to 512 despite 3 L of intravenous fluid. An arterial blood gas showed type 1 respiratory failure with a haemoglobin 98 g/L. A broad screen for infectious and non‐infectious aetiologies was performed, and a routine HIV viral load taken 4 days prior to presentation was 146 copies/mL. Anti‐retrovirals were changed to dolutegravir, efavirenz, and lamivudine due to renal failure.

**Figure 1:**
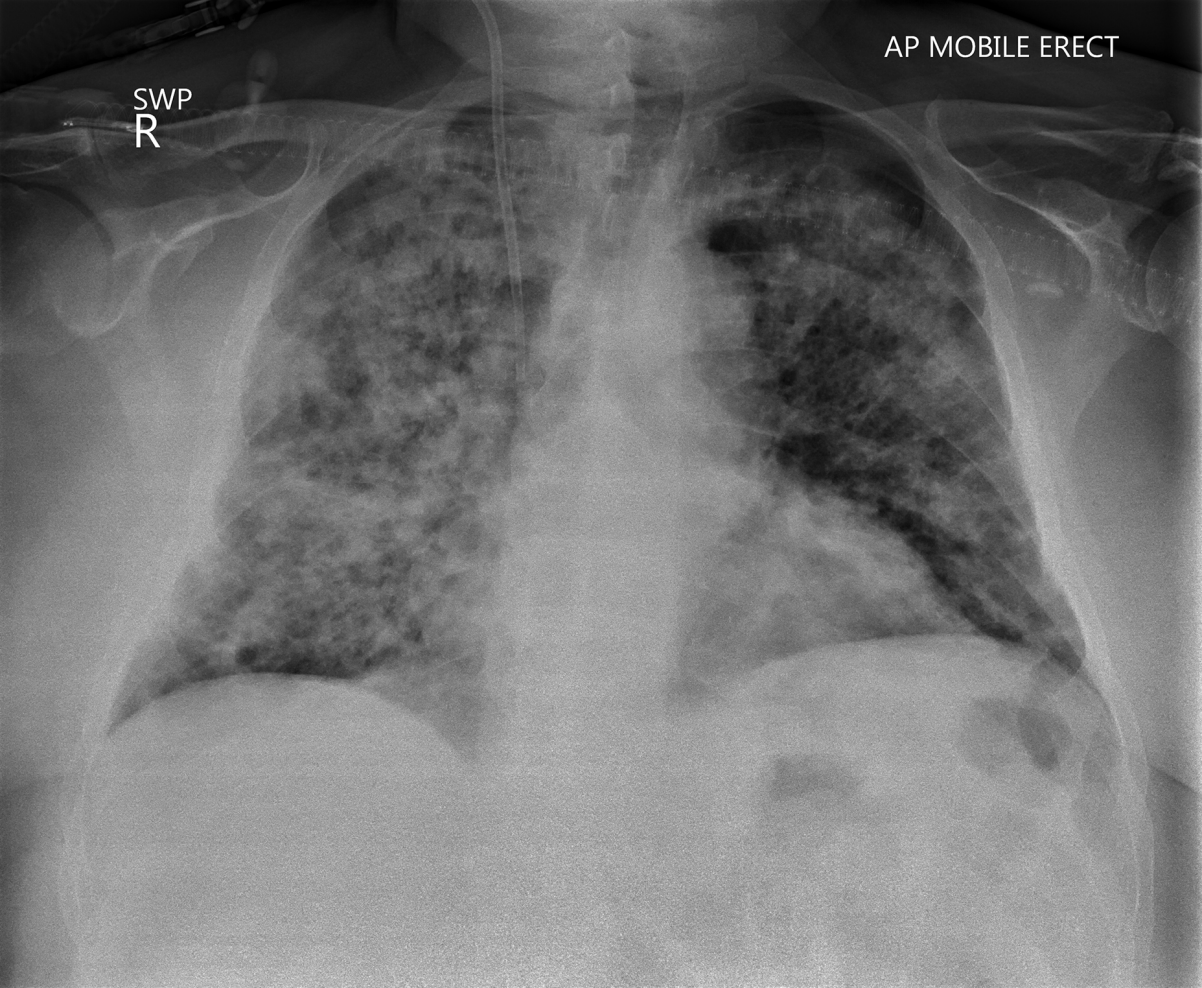
Chest radiograph at day 3 of admission. Note significant bilateral but asymmetrical infiltrates.

By day 3 his fatigue and dyspnoea had worsened without new symptoms. Repeat chest film demonstrated worsening infiltrates (Fig. 1). His oxygen requirement had increased to 50 L/min, 50% FiO_2_ via hi‐flow nasal cannulae. Dialysis was commenced with red cell transfusions as his haemoglobin was now 82 g/L. A repeat urine had >500 erythrocytes, protein 1100 mg/L, creatinine 188 g/mol, myoglobin 14 μg/L, and normal haemosiderin.

Day 4 returned a Perinuclear‐ANCA (P‐ANCA) titre >1:640 with all other diagnostic investigations unremarkable, and a provisional diagnosis of MPA was made. Treatment with intravenous cyclophosphamide (800 mg second weekly, three doses), plasma exchange (second daily, seven sessions) and methylprednisolone (500 mg daily for 3 days, then long‐course prednisolone) was started. He also received prophylactic trimethoprim/sulphamethoxazole, intravenous iron, and darbepoetin alfa.

**Figure 2:**
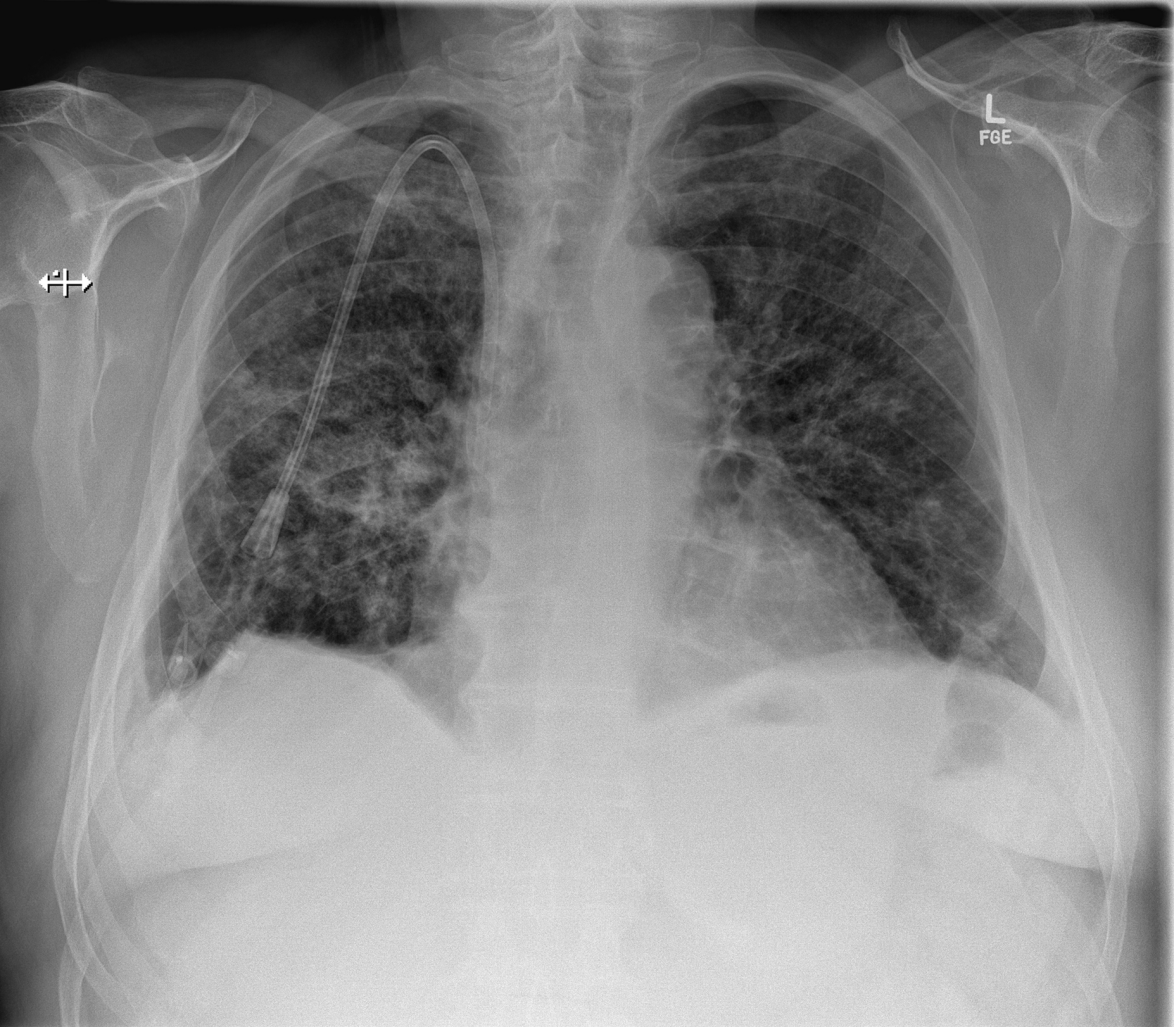
Chest radiograph at time of discharge. Significant improvement in bilateral infiltrates.

With a substantial clinical and radiological improvement (Fig. 2) he was discharged after a 29‐day stay with ongoing thrice weekly haemodialysis. Renal biopsy five days after discharge demonstrated crescentic‐class pauci‐immune necrotizing glomerulonephritis without granulomata, consistent with MPA.

## Discussion

We present a patient with symptoms and disease features of likely incidental MPA with underlying HIV infection.

Necrotizing vasculitides were first described in the mid 19th century by Kussmaul and Maier, with a series of diagnostic refinements occurring throughout the 20th century based on vessel size and histopathologic features 1.

Multiple diagnostic criteria now exist including the Chapel Hill Consensus Criteria (CHCC) and the American College of Rheumatology criteria. These diagnostic criteria attempt to differentiate vasculitides based on vessel size, organ involvement, and features, including immune complex deposition, granulomata, and ANCA positivity. More recently the Diagnostic and Classification Criteria in Vasculitis Study (DCVAS) has commenced in an attempt to create a new unifying diagnostic algorithm.

Common presenting symptoms include fever, fatigue, malaise, and arthralgias. Symptoms can present acutely with rapid deterioration or have a subacute progression, and relapses can occur after treatment onset. Sinopulmonary disease and glomerulonephritis are seen in the majority of patients, with severity varying on disease subtype 1. European League Against Rheumatism (EULAR) treatment guidelines recommend induction therapy with combination glucocorticoids and pulsed intravenous cyclophosphamide or rituximab 2.

Viral infections including HIV have been implicated in the development of vasculitis 3. The mechanism is unclear but thought to be related to inflammation, with virus‐induced immunological change, introduction of anti‐retroviral therapy, and the virus itself all playing a role 4.

It is known that HIV viral load can rise transiently during severe acute infectious illness 4, although evidence of this occurring in the setting of an auto‐inflammatory or vasculitic process is less robust. Additionally although various vasculitides have been described in the HIV‐positive population previously they more commonly manifest as an isolated cutaneous or polyarteritis syndrome rather than small vessel. A comprehensive review by Calabrese was unable to find a single documented case of small vessel vasculitis that met CHCC diagnostic criteria for MPA 5.

In summary we have presented what we believe is the first documented case of MPA as per CHCC occurring in a HIV‐positive patient. This case serves as a reminder that a patient can have many distractors, so one must always consider a broad differential diagnosis and investigate each path fully.

### Disclosure Statement

Appropriate written informed consent was obtained for publication of this case report and accompanying images.
